# Wheatgrass Juice Administration and Immune Measures during Adjuvant Chemotherapy in Colon Cancer Patients: Preliminary Results

**DOI:** 10.3390/ph13060129

**Published:** 2020-06-23

**Authors:** Adva Avisar, Miri Cohen, Rina Katz, Talia Shentzer Kutiel, Anat Aharon, Gil Bar-Sela

**Affiliations:** 1 The Graduate Studies Authority, University of Haifa, Haifa 31000, Israel; advah7@gmail.com; 2 School of Social Work, University of Haifa, Haifa 31000, Israel; mcohen2@univ.haifa.ac.il; 3 Clinical Immunology and Tissue Typing Lab, Rambam Medical Center, Haifa 31000, Israel; r_katz@rambam.health.gov.il; 4 Division of Oncology, Rambam Health Care Campus, Haifa 31000, Israel; T_SHENTZER@rambam.health.gov.il; 5 Hematology and Bone Marrow Transplantation, Sourasky Medical Center, Tel Aviv 6423906, Israel; a_aharon@yahoo.com; 6 Rappaport Faculty of Medicine, Technion-Israel Institute of Technology, Haifa 31000, Israel; 7 Cancer Center, Emek Medical Center, 21 Yitzhak Rabin Blvd., Afula 1834111, Israel

**Keywords:** colon cancer (CC), adjuvant chemotherapy, wheatgrass, immune measures

## Abstract

Adjuvant chemotherapy is recommended in high-risk stage II–III colorectal cancer (CC). We examine the effect of daily wheatgrass juice (WGJ) intake in addition to chemotherapy on immune parameters, including IL-6, IL-8, IL-10, IL-12, and white blood cells (WBCs) among CC patients. In a controlled prospective trial, 100 stage II–III CC patients were enrolled. According to patient preference, they were divided into two subgroups, control group and intervention group, 50 patients each, all of whom received the same standard postoperative adjuvant chemotherapy, plus consumption of 60 cc WGJ daily in the intervention group. Blood samples were collected at baseline (T0) and upon treatment termination, 5–6 months later (T1). Cytokine concentrations were assessed using ELISA kits. Anti-inflammatory cytokine IL-10 concentrations were significantly higher in the WGJ group than in the control group at T1. The decline in WBC counts between T0 and T1 was significantly lower in the WGJ group. No significant differences were observed in IL-6, IL-8, and IL-12 concentrations between the study groups. The higher levels of IL-10 and the attenuating of WBC decline during chemotherapy may constitute preliminary evidence of the beneficial effects of WGJ on immune parameters, when given as a supplement to standard care. In light of these preliminary results, WGJ supports immunological parameters during adjuvant chemotherapy. Nevertheless, future studies are needed in order to translate those results to clinical recommendations for cancer survivors.

## 1. Introduction

Colorectal cancer (CC) is the third most common type of cancer, accounting for 10% of all cancer cases. It is the second most prevalent type in terms of the number of individuals living with cancer five years after diagnosis, worldwide [1]. Six months of adjuvant chemotherapy with 5-fluorouracil and leucovorin (5-FU/LV) or capecitabine and oxaliplatin is the current worldwide standard of care for patients with high risk stage II or III colon cancer following curative surgery [2]. The high incidence of CC cancer patients and the increased rate of survivors raise an urgent need for studies involving nutritional interventions aimed at the immune system’s functions and alleviating chemotherapy’s adverse effects.

Cytokines are a diverse group of intracellular messengers. They orchestrate and regulate the human immune and inflammatory responses and maintain a proper balance among the various immune cell types [3,4]. Some cytokines are usually considered as pro-inflammatory (e.g., IL-6, IL-8), and others are generally considered as anti-inflammatory (e.g., IL-10). In healthy conditions, inflammatory processes are self-limiting and self-resolving [5]. However, unsuccessful resolution may lead to chronic, self-promoting inflammation, characterized by excessive tissue damage [6]. Thus, although the cytokine mediated response is essential, exaggerated production of pro-inflammatory cytokines and chronic inflammation is associated with progression and mortality among cancer patients [4,7]. The anti-inflammatory cytokines are a series of immune-regulatory molecules that control the pro-inflammatory cytokine response. Thus, they limit sustained or excessive inflammatory reactions [8,9].

Leukopenia, a low white blood cell (WBC) count, is commonly caused by chemotherapy [10]. In particular, neutropenia and febrile neutropenia are the most frequently observed dose-limiting toxicities associated with chemotherapy use among cancer patients [11]. Two studies suggested inverse correlation between pro-inflammatory cytokines and WBC counts during chemotherapy. It has been reported that pro-inflammatory cytokine IL-6 levels rise in patients with chemotherapy-induced myelosuppression [12]. Similarly, another study reported that IL-6 and TNF-α increased almost at the same time that the numbers of neutrophils decreased after chemotherapy infusion [13]. Consistent with the current study hypothesis, WGJ intake may lower systemic inflammation, and the change (Δ) in WBC count during chemotherapy was expected to be milder in the WGJ intervention group.

A wide range of health benefits have been attributed to wheatgrass, the young grass of the common wheat plant, Triticum aestivum. Its components include chlorophyll, flavonoids, and a number of vitamins (C, E, A), minerals, amino acids, and vital enzymes [14,15]. Forms of wheatgrass include fresh juice, frozen juice (used in the present study), tablets, and powders, with compositions varying according to their production processes, as well as to the growing conditions of the wheatgrass [16].

Laboratory in vitro studies, mostly using the fermented wheat germ extract, have demonstrated anticancer potential: antiproliferative effects were observed in different cancer cell lines [15,17,18]. Further in vitro studies have identified apoptosis as a possible mechanism [19,20,21,22]. In animal experiments, wheatgrass demonstrated benefits in cancer prevention and as an adjunct to cancer treatment, as well as benefits to immunological activity and oxidative stress [16]. Clinical trials show that wheatgrass may induce synergistic benefits to chemotherapy and may attenuate chemotherapy-related side effects [16]. For example, in a study with colon cancer patients, after six-month supplementation of fermented wheat germ extract to anticancer treatments, lower recurrences of metastatic disease and mortality were reported in the intervention group [23]. Frozen WGJ was used in a previous clinical study, together with adjuvant chemotherapy given to breast cancer patients [24]. In this matched pairs study, patients received WGJ daily during adriamycin-based adjuvant chemotherapy, while matched patients received only chemotherapy. The most important effect observed was a reduction in neutropenic fever events and in neutropenic infections [24]. However, there is only a limited number of intervention studies with wheatgrass among cancer patients, and most of these are small.

Overall, possible associations between WGJ consumption and improved immune and inflammatory measures may imply beneficial effects of wheatgrass on the disease course, although there is a lack of established empirical knowledge concerning the subject. Therefore, the current study aimed to examine the possible effects of daily WGJ intake on immune measures: pro- and anti-inflammatory cytokines IL-6, IL-8, IL-10, and IL-12 and WBC change (Δ) during chemotherapy.

## 2. Results

Baseline demographics, disease stage, chemotherapy regimen, background diseases, and anticoagulants drugs were balanced across the study population. Consequently, there were no significant differences between the two groups regarding the background variables (Table 1). Patients’ mean age was 63.2 years for the WGJ intervention group and 60.8 years for the control group. Respectively, 72% and 60% in the WGJ group and the control group were males. For both groups, the mean number of years of education was 14, the majority of the participants were currently married or living with a partner, and around 30% continued to work at baseline (T0). Most participants reported themselves as current or past cigarette smokers, and about 40% reported moderate or high physical activity (moderate physical activity included 40 min walking three times a week). In both groups, the vast majority of patients was diagnosed with stage III CC, the most frequent chemotherapy protocol was capecitabine and oxaliplatin, and the average number of background diseases was slightly above 1.

As part of the follow-up during the study, participants were asked regarding coping with chemotherapy, how well they were handling daily WGJ consumption, and whether any difficulties had arisen. Thus, follow-up of patients in the WGJ intervention group was maintained throughout the study, allowing for assessing the persistence of juice consumption and for responding to questions. Patients demonstrated good adherence to daily juice intake (60 cc) during the study period. In general, patients viewed WGJ consumption as an integral part of treatment, along with the medication routine. Hence, consumption was usually continuous during the treatment period (according to patients’ reports, adherence to daily 60 cc WGJ intake was estimated to be over 90%). In a few cases (*n* = 4), a few days of pause in WGJ consumption were reported due to increased nausea, cold sensitivity, etc., following the chemotherapy infusion. However, after several days’ break, the intake was resumed. In two other cases, a break in consumption for several days due to a temporary departure from the country and the resumption of intake upon return was reported.

Similar mean concentrations of cytokines IL-6, IL-8, and IL-12 at T0 and T1 were found in both study groups, with no statistically significant differences (Table 2). In contrast, there was a significant difference between the groups in the anti-inflammatory cytokine IL-10 concentration; it was significantly higher in the WGJ intervention group compared to the control group at T1. Moreover, a significantly higher monocyte count was observed in the WGJ intervention group at T1 (Table 2). However, no significant differences were observed in the remaining WBC subpopulations between study groups at T1. In addition, the difference or change (Δ) between T0 and T1 in WBC counts was also tested and compared between the groups (differences were calculated using subtraction of WBC counts at T0 from WBC counts at T1) (Table 2). The data indicated that the mean difference of total WBC counts was significantly greater in the control group, compared with the intervention group (*p* < 0.05). Similarly, there was a trend (*p* = 0.07) for a greater mean difference of neutrophil counts in the control group. Hence, the decrease in total WBC and neutrophil counts during chemotherapy was less in the intervention group, with similar mean differences of lymphocyte and monocyte counts in both groups.

Table 3 presents the results of four multivariate models conducted to predict the blood concentrations (pg/mL) of the four pro- and anti-inflammatory cytokines. A linear mixed effect regression model (LMM) was used. The predictor variables included group variable, time, and interaction of time with the group variable, as mentioned earlier. The relevant background variables for each of the outcome variables were included as well. The group variable had a significant positive effect on IL-10; the WGJ intervention group was associated with a higher IL-10 blood concentration. Time had no effect on the cytokine concentration, and the group variable had no effect on IL-6, IL-8, and IL-12 concentrations.

Table 4 presents the results of four LMM models conducted to predict total WBC, neutrophils, lymphocytes, and monocytes (K/µL). The analysis showed that time had a significant negative effect on total WBC, neutrophils, and lymphocytes (but not on monocytes) counts. Trends (*p* = 0.07) were observed for statistically significant interactions of time with the group variable in both models that predicted total WBC and neutrophil counts, indicating a milder decline in these counts during chemotherapy in the WGJ intervention group. These interactions reveal that the change in WBC counts from baseline throughout chemotherapy and the change in neutrophils in particular were different between the study groups. In addition, the group variable had a significant positive effect on lymphocyte and monocyte counts: the WGJ intervention group was associated with higher counts of lymphocytes and monocytes during chemotherapy.

### Clinical Outcomes

The study was not planned to show differences in clinical outcomes. Nevertheless, the main outcomes are reported (Table 5). Diarrhea was observed in both study groups without significant difference (63% did not have diarrhea; 18% reported diarrhea grade I–II and 19% grade III). No significant difference in occurrence of diarrhea grade ≥III during the chemotherapy treatment period was observed with the addition of daily WGJ consumption (16% WGJ group and 22% in the control, *p* = 0.47). Grade ≥II nausea and vomiting was less reported in the WGJ group; three patients (6%) compared to eight (16%) in the control arm (0.12). The number of hospitalizations during the treatment period was reported for 25% of the patients, without significant difference of occurrence between the control and WGJ groups (22% in the WGJ and 28% in the control). Two patients died during the treatment period due to intolerable toxicity (one in each group). Median follow-up time was 22 months for both groups.

With a median follow-up of 24 months, the number of patients with recurrent disease was 23, with 14 in the WGJ group compared with 9 in the control group (*p* = 0.24). Eleven patients had died at the time of analysis, three in the WGJ group (one due to toxicity and two due to metastatic disease) and eight in the control group (one due to toxicity, one from multiple organ dysfunction, five due to metastatic disease and one after four years from unknown causes).

Overall survival (OS) in the 1st, 2nd, and 3rd year was 96%, 87%, and 84%, respectively, in the control group and 98%, 96%, and 96%, respectively, in the WGJ group (*p* = 0.14). There was no statistically significant difference in all clinical parameters. Although the OS result favored wheatgrass supplementation, this may also have partly related to a higher percentage of control patients with Stage III CC (94% versus 80%) at baseline.

## 3. Discussion

Only a limited number of intervention studies with wheatgrass were conducted among cancer patients previously [23,24,25,26,27]. The relationship between wheatgrass consumption and inflammation measures has not been studied at all, to the best of our knowledge. The current study indicates for the first time that anti-inflammatory cytokine IL-10 concentration was higher in the WGJ intervention group compared with the control group at the end of the chemotherapy period. Further, the decrease in WBC and neutrophil counts during the chemotherapy treatment period was attenuated with WGJ consumption.

Oncology treatment, chemotherapy, and cancer surgery may induce and enhance inflammation [28,29]. In turn, increased levels of inflammation and pro-inflammatory cytokines may enhance disease symptoms, including pain, cognitive dysfunction, dyspnea, fatigue, and depression [30,31]. Chronic inflammation can promote all stages of tumorigenesis, including DNA damage, limitless replication, sustained angiogenesis, and metastasis [32]. Accordingly, studies indicate that pro-inflammatory cytokines may induce angiogenesis and metastasis and promote tumor growth [33]. In contrast, there are studies which imply that high levels of anti-inflammatory cytokines may have a protective effect against cancer development [34,35]. However, these relationships are not yet established. In the current study, we considered the associations between WGJ intake and certain pro- and anti-inflammatory cytokines.

According to a few intervention studies, a number of dietary supplements and herbs may affect levels of cytokines involved in inflammation among cancer patients [36,37]. For example, it was found that omega-3 can promote the release of IL-10 [37]. These results resemble the findings of the current study, which indicate higher levels of IL-10 in the WGJ intervention group. However, other studies have not demonstrated similar results [38,39,40]. Overall, most prior studies were conducted with small samples, and short intervention periods [36,37,41,42,43].

Evidence of wheatgrass anti-inflammatory properties was obtained from one animal study and from two small human intervention studies that demonstrated alleviation of inflammatory disease symptoms [44,45,46]. Wheatgrass has been shown to alleviate the symptoms of atopic dermatitis in a mice animal model [46]; and two intervention studies demonstrated symptom relief following consumption of wheatgrass in patients with arthritis and ulcerative colitis (inflammatory bowel disease) [44,45]. The findings of the current research are supported by these studies, and may also offer a mechanism for explaining the results described above: higher concentrations of anti-inflammatory cytokine IL-10 due to wheatgrass intake may explain, at least partially, the mechanism underlying alleviation of these inflammatory disease symptoms [44,45,46].

The findings of the present study can also be explained on the basis of the high content of anti-inflammatory components in wheatgrass. Hence, the increased concentration of anti-inflammatory cytokines IL-10 found in the WGJ intervention group can be elucidated by the high content of chlorophyll, flavonoids, and superoxide dismutase in wheatgrass [47]. Chlorophyll, which constitutes one of the major components of wheatgrass, may contribute to the treatment of various inflammation-related diseases, such as dengue [48], cholangiocarcinoma [49], allergic rhinitis [50], and acne vulgaris [51]. Further, it is conceivable that wheatgrass may also reduce oxidative stress induced by inflammation. This is due to a high antioxidant content, as well as antioxidant effects, that were verified in prior studies [27,47,52]. Overall, wheatgrass intake may contribute to decreased inflammation as well as decreased oxidative stress induced by inflammation.

Some clues toward trends of decreased levels of pro-inflammatory cytokines in the WGJ intervention group can be found in the current study. For instance, a steeper decrease in the pro-inflammatory cytokine IL-8 concentration during treatment occurred in the intervention group compared with the control group (Table 2). Moreover, the insignificant results regarding the inflammatory cytokines may be explained due to limited sample size and high dispersion: a wide range of cytokine levels. In addition, it can be hypothesized that the significant difference in the anti-inflammatory cytokine IL-10, along with the non-significant differences in the inflammatory cytokines, are related to the regulatory role of IL-10. These results may indicate that the role of IL-10 is reflected through inflammation reduction in the long term rather than in the short term.

Although the chemotherapy protocols that are used in the adjuvant chemotherapy treatment for CC patients rarely brought about severe neutropenia or leucopenia, those side-effects are the most frequently observed dose-limiting toxicities associated with chemotherapy [11]. Severe neutropenia (<0.5 K/uL) is the main risk factor for infections [11]. Therefore, it is important to consider therapies that may provide support for the immune system and specifically for WBC counts during chemotherapy. WGJ may have such potential. In the current study, the interactions of time with the group variable reveal that the change in WBC counts from baseline throughout chemotherapy, and the change in neutrophils in particular, was different between the study groups. Thus, although the WBC counts decreased in both groups during therapy, the current study suggests that the decrease in WBC and neutrophils was milder in the intervention group.

Intervention studies of the effects of different medicinal plants on cancer patients during chemotherapy have demonstrated higher WBC counts in the intervention groups, similar to the current study [53,54,55]. Several animal and human studies specifically conducted with wheatgrass also imply a beneficial effect of wheatgrass on WBC counts or adverse effects associated with WBC decline [56,57]. A reduction in neutropenic fever events and in neutropenic infections due to intake of wheatgrass was reported among breast cancer patients [24] and pediatric patients [25]. In conclusion, the findings of the current as well as previous studies suggest that wheatgrass may benefit WBC count or the adverse effects associated with a low count.

Higher lymphocyte counts in the intervention group were also found in the current study. Prior studies in medicinal plants (mainly used in traditional Chinese medicine) have also demonstrated higher counts of lymphocytes of different types in the intervention group among cancer patients [53,55,58,59], although results are inconclusive [39]. It can be assumed that a beneficial effect of WGJ on lymphocyte counts, observed in the current study, may decrease lymphopenia among patients. Previous studies have revealed that severe lymphopenia (<0.5 K/uL) due to chemotherapy and radiation therapy was associated with lower survival and disease progression in various cancers [60,61], including postoperative colorectal cancer patients [62], and during adjuvant chemotherapy [63], similar to patients included in the present study.

The present study also indicates higher counts of monocytes (phagocytes cells that protect the body against infection) in the intervention group. Monocyte counts increased during chemotherapy in both groups, with a steeper increase in the intervention group. IL-10, which regulates and restrains the inflammatory process, is usually secreted by immune cells, particularly monocytes [64]. Therefore, the fact that the monocyte counts were higher in the intervention group is consistent with higher concentrations of IL-10 in this group at T1.

A possible elucidation for the higher monocyte and lymphocyte counts, milder decline in WBC, and a higher concentration of IL-10 observed in the WGJ intervention group emerges from a study examining the effect of oligosaccharides isolated from wheatgrass on human peripheral blood mononuclear cells [65]. According to this study, wheatgrass-derived oligosaccharides have immunity-modulating properties; it was found that they activated various immune cells, including T cells, NK cells, and monocytes. In particular, they directly modulated monocytes through Toll-like receptor 2 (TLR-2). In addition, upregulated secretions of Th1 cytokines in human peripheral blood mononuclear cells were also observed in the presence of the oligosaccharides [65]. Hence, this in vitro study revealed a beneficial effect of wheatgrass upon the immune system in line with the results of the present study.

In summary, differences were observed in all WBC subpopulations examined between the two study groups. Both current and prior studies suggest that wheatgrass has the potential to serve as a supportive therapy during chemotherapy, with a protective effect from leucopenia and the adverse effects associated with it.

As mentioned earlier, the study was not planned to show differences in clinical outcomes. However, several chemotherapy side-effects were reported. Numerically, fewer patients in the WGJ group had nausea and vomiting or severe diarrhea. Nevertheless, with respect to hospitalizations during treatment, no significant differences were found between the groups. The chemotherapy protocol based on 5-FU and oxaliplatin used for colon cancer usually does not cause severe neutropenia. Although relief from hematologic side effects (decreased WBC counts) was demonstrated in the present study, it was not translated to clinical outcomes.

The results of the current study may indicate a survival benefit for patients in the WGJ group; this is in line with the beneficial effect of WGJ on immune functions demonstrated in this study. However, this benefit was not significant (*p* = 0.14). Based on the difference in survival after 24 months of follow-up seen in the current study, the number of patients needed to be treated is 268 in order to reach significance. This study is under-powered to reach any conclusion regarding survival benefits of WGJ during the chemotherapy period. It may also logically hypothesize that the most significant effect of WGJ on disease course and survival is during juice intake, and not necessarily a considerable period of time after cessation of consumption. Therefore, in order to answer the question of survival advantage, a larger study with a longer period of WGJ consumption is needed.

## 4. Methods

### 4.1. Patients and Procedure

One hundred patients were recruited consecutively between 6 March 2014 and 11 July 2017. All procedures performed in studies involving human participants were in accordance with the ethical standards of the institutional and/or national research committee and with the 1964 Helsinki declaration and its later amendments or comparable ethical standards. All participants signed an informed consent form for inclusion prior to their participation. The study was approved on 9 December 2013, by the Institutional Review Board of Rambam Health Care Campus (RHCC) in Haifa, Israel (approval no. 0153-13) (NIH number NCT01991080). The present study is part of a larger research project conducted with regard to WGJ consumption in relation to extracellular vesicles (EVs) and psychological variables. The inclusion criteria were as follows: patients aged 18+, diagnosed with CC stages II–III toward adjuvant chemotherapy following curative surgery. Exclusion criteria were: patients with an infectious disease (hepatitis B), or with difficulty understanding the Hebrew language. However, the number of patients discharged due to these causes was low (*n* = 5). The selection of suitable candidates for the study was conducted through the hospital’s computerized system. Letters were sent to suitable candidates to inform them of research being conducted in the Division of Oncology at RHCC. A week later, the patients were telephoned to suggest participation in the study, as well as to provide information and explanation regarding the study and to answer questions. Response rate was 70.5%.

Patients who were interested in consuming WGJ were admitted to the WGJ intervention group. Patients who preferred not to consume WGJ were asked to donate blood samples for the purpose of the study, and those who agreed were entered into the control group. The chemotherapy period lasted for 5–6 months for all patients who did not stop treatment due to toxicity (the dropout rate was 5%, and 25% of patients did not complete the chemotherapy treatment period). The period of WGJ intake was identical to the chemotherapy period for the relevant group. Consistent follow-up with all patients was maintained throughout the study period (control and intervention groups). Monitoring and communication with participants were conducted equally for both groups. The follow-up was usually maintained via a personal frontal conversation when patients arrived at RHCC to receive chemotherapy treatment or to visit the attending physician, once every two to three weeks. If this was not possible, a telephone call was made instead. During these follow-up conversations, it was possible to monitor patients’ well-being, coping with chemotherapy, difficulties that arose, and adherence to WGJ intake.

### 4.2. Study Intervention

In the current study, we used pure natural WGJ without any processing or additives, supplied by a single agriculturist. The grass was grown on unified compost, consisting entirely of organic manure. Wheat seeds were germinated on trays that contain the compost throughout the year. There was an automatic constant irrigation during the growth. Harvest took place whenever the wheat sprouts reached a length of about 15–20 cm (when their nutritional values are at peak). The content of nutritional components of the wheatgrass grown in this method does not change throughout the year (although the sugar content is slightly higher on sunny days compared to cloudy days). Squeezing was always cold press-done by a special slow rpm juicer. As a result, the grass, as well as the juice, stays cool throughout the process, and nutritional values are preserved. Moreover, squeezing was carried out immediately before freezing in order to maintain these nutritional values. Each patient in the intervention group received a monthly supply of frozen WGJ, divided into daily rations of 60 cc, during the chemotherapy treatment period (5–6 months). The juice was kept in a freezer, and the participants were asked to drink one dose every morning on an empty stomach.

### 4.3. Blood Samples

Blood samples were obtained from each patient before the first chemotherapy cycle (baseline, T0) and after completion of the chemotherapy treatment 5–6 months later (post intervention, T1). Briefly, 15 mL of peripheral venous blood were drawn from study participants into ethylenediaminetetraacetic acid (EDTA) tubes; plasma was obtained after two centrifugations (15 min, 1500 g) within an hour of collection and frozen at −80 °C. Samples were used to assess pro- and anti-inflammatory cytokine concentrations using appropriate kits (Human ELISA MAX Deluxe, BioLegend, San Diego, CA, USA).

### 4.4. Cytokines Assessment

For cytokine quantification, the following kits were used: Human IL-6 ELISA MAX Deluxe, BioLegend, San Diego, CA, USA. cat # 430505; Human IL-8 ELISA MAX Deluxe, BioLegend, San Diego CA, USA. cat # 431505; Human IL-10 ELISA MAX Deluxe, BioLegend, San Diego, CA, USA. cat # 430605; Human IL-12 p70 ELISA MAX Deluxe, BioLegend, San Diego, CA, USA. cat # 431705 (https://www.biolegend.com/Files/Images/media_assets/pro_detail/datasheets/430504_V02.pdf).

Evaluation of pro- and anti-inflammatory cytokines (IL-6, IL-8, IL-10, IL-12) was performed according to the manufacturer’s instructions. Briefly, a day prior to the experiment, the plates were coated with a primary (Capture) antibody (100 µL; dilution, 1:200) and incubated overnight at 4 °C. On the experiment day, washing was performed four times (and performed later, after each step), and the plates were blocked. Standard and samples (100 µL) were added in duplicates to the wells containing capture antibodies and incubated for two hours with gentle shaking (the samples were not diluted). Biotinylated detection antibody (100 µL; dilution, 1:200) was then added to the wells. After 60 min of incubation, avidin–HRP (horseradish peroxidase) complex was added. Thirty minutes later, the substrate (tetramethylbenzidine substrate solution) was added, and the plates were incubated in the dark. At this point, a blue dye was formed, which was proportional to the concentrations of each of the cytokines in the blood samples. The reaction was stopped by adding 100 μL of acid solution to each well, and the plates were read at 450 nm.

Clinical data at T0 and WBC data at T0 and T1 were collected from the patient’s medical record, as these are routine tests for patients receiving this chemotherapy regimen. Apart from total WBC, subpopulations of neutrophils, lymphocytes, and monocytes were also collected and studied. Demographic data were collected using a self-report demographic questionnaire.

### 4.5. Statistical Analysis

Statistical analyses were performed using SPSS (version 23) software for Windows. The chi square test and the independent *t* test were used to determine the difference between patients’ demographic and clinical characteristics in both groups. Differences between the study groups regarding the immune variables at T0 and T1 were calculated using the independent *t* tests. In order to test these differences while adjusting for background variables, a linear mixed effect regression (LMM) was used.

The main advantage of LMM lies in the fact that the analysis includes the data of all study participants, including patients who had not completed chemotherapy (according to intention to treat approach). From the clinical and demographic variables, only those variables that were correlated significantly with the outcome variables were entered into the LMM models and adjusted for. They were entered to the models as covariates together with the group variable, the time variable, and the interactions between time and group. The group variable examined whether the intervention had an effect on the immune variables, compared with the control group; the time variable examined whether time had an effect on the immune variables; and the interaction between time and group tested whether the change over time in the immune variables differed between the two groups. The background variables were added to the models in order to adjust for their effect on the relationships between group and time variables and the immune variables.

Background variables that were significantly associated with the outcome variables (immune measures) were: education (for IL-6); gender, education, background diseases (for IL-8); gender (for IL-10); education, smoking (for IL-12); smoking (for WBC); employment (for lymphocytes); gender, disease stage (for monocytes). No significant associations between neutrophils and background variables were found.

*p* Values of 0.05 were considered statistically significant. However, a statistical significance level of *p* < 0.1 was presented as well. This is due to the difficulty of reaching statistical significance when the dependent variable is biological, similar to previous studies [53,66].

The estimated sample size was calculated according to Cohen’s equation; in a regression model with six independent variables, in order to achieve a medium effect size, at a significance level of 0.05, and a statistical power of 0.80, a sample of 100 participants was required [67].

## 5. Study Limitations

The main limitation of this study is the non-random allocation into intervention and control groups, based on self-selection. This could result in patient-related bias (volunteer bias). However, no statistically significant differences were found in baseline demographic and clinical characteristics between the study groups. Moreover, the baseline characteristics that were significantly associated with the outcome variables (immune measures) were added to the LMM regression models in order to adjust for their effect on the relationships between WGJ intervention, time, and the immune outcome variables. Further, the same characteristics that motivated patients in the study sample to choose WGJ consumption most likely resemble the characteristics that would motivate patients in the general population to consume WGJ (e.g., tendency toward healthy diet, health habits). It is therefore plausible to assume that the findings of the current study would apply to the general population of CC patients, despite self-selection. In addition, although the study was controlled, a placebo effect was not tested.

## 6. Conclusions

This is a preliminary study devised to examine the possible effects of WGJ on the immune system. Our findings in relation to the anti-inflammatory cytokine IL-10, as well as WBC counts during chemotherapy, constitute preliminary evidence highlighting the beneficial effects of WGJ on immune parameters, when given as a supplement to standard care. There is preliminary evidence, both from the current and previous studies, that wheatgrass has the potential to mitigate a number of different chemotherapy-induced damages. Moreover, wheatgrass constitutes no adverse effects comparable to chemical drugs. Hence, it has a potential to be used as a cheap, non-toxic supplement to standard chemotherapy. The present study, therefore, contributes to the creation of evidence-based recommendations for the use of WGJ among CC patients during chemotherapy.

## Figures and Tables

**Table 1:** Background characteristics of the participants by group.

Variable	Intervention	Control		*p*
	(*n* = 50)	(*n* = 50)		
Age (years), M (SD)	63.2 (10.2)	60.8 (10.4)	*t* = −1.16	0.25
Gender, N (%) ^a^				
Male	36 (72.0)	30 (60.0)	χ^2^ = 1.60	0.21
Female	14 (28.0)	20 (40.0)		
M (SD) Education (years),	14.3 (4.9)	14.0 (3.8)	*t* = −0.34	0.74
Familial status, N (%)				
Married/cohabiting	40 (80)	37 (74.0)	χ^2^ = 3.38	0.34
Not married/cohabiting	10 (20.0)	13 (26.0)		
Employed, N (%)	13 (26.5)	16 (35.5)	χ^2^ = 8.23	0.22
Smoking, N (%)				
Current	6 (13.0)	12 (25.5)	χ^2^ = 2.44	0.30
Past	19 (41.3)	18 (38.3)		
Never	21 (45.7)	17 (36.2)		
Physical activity, N (%)				
Low	24 (54.5)	24 (61.5)	χ^2^ = 0.53	0.77
Medium	15 (34.1)	12 (30.8)		
High	5 (11.4)	3 (7.7)		
Disease stage, N (%)				
Stage II	10 (20.0)	3 (6.0)	χ^2^ = 4.35	0.11
Stage III	40 (80.0)	47 (94.0)		
Chemotherapy regimen, N (%)				
Xeloda (Capecitabine)	11 (22.0)	3 (6.0)	χ^2^ = 6.21	0.10
Xelox (Capecitabine, Oxaliplatin)	35 (70.0)	42 (84.0)		
Folfox (5-fluorouracil, Leucovorin, Oxaliplatin)	4 (8.0)	4 (8.0)		
Number of background diseases, N (SD)	1.04 (1.16)	1.10 (1.23)	*t* = −0.25	0.80
Anticoagulant treatment, N (%)	12 (24.0)	14 (28.0)	χ^2^ = 0.21	0.65

M = mean, SD = standard deviation, *p* = *p* Value, ^a^ percentages are calculated from the total respondents for each item.

**Table 2:** Means (SD) of cytokine concentrations ^a^ white blood cell (WBC) concentrations ^b^ and differences ^c^ in WBC concentrations, by group at each assessment time.

	Intervention *n* = (50)			Control *n* = (50)				
	M	SD	CI	M	SD	CI	*t* (df)	*p*
**IL-6**								
Baseline (T0)	21.16	8.51	18.98, 23.64	19	9.63	16.45, 21.92	−1.17 (95)	0.25
Post intervention (T1)	21.35	8.33	18.90, 24.07	20.12	9.25	16.88, 23.67	−0.56 (62)	0.58
**IL-8**								
Baseline (T0)	21.93	14.06	18.07, 25.90	17.59	13.56	13.84, 21.28	−1.55 (95)	0.13
Post intervention (T1)	16.33	11.79	13.07, 20.21	16.08	13.53	11.25, 21.77	−0.08 (62)	0.94
**IL-10**								
Baseline (T0)	4.62	2.66	3.89, 5.41	4.42	4.34	3.41, 5.84	−0.28 (95)	0.78
Post intervention (T1)	5.14	2.55	4.33, 5.93	3.8	2.18	2.95, 4.59	−2.19 (62)	0.03
**IL-12**								
Baseline (T0)	26.51	15.2	22.16, 30.77	23.66	13.19	20.29, 27.86	−0.98 (95)	0.33
Post intervention (T1)	22.57	12.71	18.67, 26.91	23.45	15.33	18.24, 29.24	0.25 (63)	0.8
**WBCs**								
Baseline (T0)	6.77	1.56	6.34, 7.22	6.92	2	6.38, 7.52	0.42 (92)	0.67
Post intervention (T1)	6.2	2.07	5.54, 6.99	5.61	1.71	5.01, 6.20	−1.27 (65)	0.21
**Neutrophils**								
Baseline (T0)	3.78	1.28	3.42, 4.17	4.26	1.37	3.89, 4.68	1.76 (91)	0.08
Post intervention (T1)	3.29	1.68	2.80, 3.87	3.09	1.3	2.68, 3.57	−0.56 (65)	0.58
**Lymphocytes**								
Baseline (T0)	2.01	0.74	1.80, 2.20	1.87	0.94	1.62, 2.15	−0.75 (89)	0.46
Post intervention (T1)	1.76	0.69	1.54, 1.98	1.61	0.75	1.36, 1.86	−0.86 (64)	0.4
**Monocytes**								
Baseline (T0)	0.68	0.23	0.61, 0.74	0.6	0.22	0.54, 0.67	−1.60 (89)	0.11
Post intervention (T1)	0.88	0.38	0.76, 1.01	0.71	0.26	0.63, 0.80	−2.15 (64)	0.04
**WBC difference ^a^**	−0.48	2.26	−1.25, 0.32	−1.48	1.63	−2.08, −0.91	−2.04 (63)	0.046
**Neutrophil difference**	−0.38	1.86	−0.97, 0.23	−1.13	1.38	−1.62, −0.65	−1.82 (63)	0.07
**Lymphocytes difference**	−0.26	0.54	−0.46, 0.10	−0.42	0.49	−0.60, −0.24	−1.17 (59)	0.25
**Monocytes difference**	0.17	0.37	0.05, 0.31	0.09	0.24	0.00, 0.18	−1.00 (59)	0.32

M = mean, SD = standard deviation, CI = confidence interval, *p* = *p* Value, ^a^ Units of cytokine concentrations: pg/mL, ^b^ Units of WBC cell concentrations: K/uL, ^C^ changes (Δ) in WBC between baseline (T0) and treatment termination (T1).

**Table 3:** Linear mixed effect regression (LMM) models for predicting cytokine concentrations (pg/mL).

	IL-6 (*n* = 90)				IL-8 (*n* = 90)				IL-10 (*n* = 97)				IL-12 (*n* = 87)			
	B	SE	95% CI	*p*	B	SE	95% CI	*p*	B	SE	95% CI	*p*	B	SE	95% CI	*p*
Time ^a^	−0.03	2.04	−4.10, 4.05	0.99	4.22	3.43	−2.60, 11.05	0.22	0.64	0.62	−0.61, 1.88	0.31	2.68	3.82	−4.92, 10.28	0.49
Group ^b^	1.63	2.41	−3.19, 6.46	0.50	0.95	3.53	−6.13, 8.03	0.79	1.28	0.63	0.02, 2.55	0.046	1.28	3.83	−6.39, 8.95	0.74
Time*Group ^c^	−0.40	2.64	−5.67, 4.87	0.88	1.58	4.43	−7.24, 10.41	0.72	−1.17	0.83	−2.84, 0.51	0.17	0.96	5.04	−9.07, 11.00	0.85
Education (years)	0.34	0.19	−0.04, 0.71	0.08	0.70	0.25	0.21, 1.19	0.01	-	-	-	-	0.43	0.24	−0.05, 0.90	0.08
Gender	-	-	-	-	2.37	2.34	−2.28, 7.02	0.31	0.55	0.57	−0.60, 1.69	0.34	-	-	-	-
Smoking	-	-	-	-	-	-	-	-	-	-	-	-	8.26	2.79	2.70, 13.82	0.00
Background Diseases	-	-	-	-	−2.81	1.03	−4.85, −0.77	0.01	-	-	-	-	-	-	-	-

Abbreviations: B = Estimate of fixed effect, SE = standard error, ***p*** = *p*-Value, C = confidence interval, ^a^ Time = T0, ^b^ Group = WGJ intervention group, ^c^ the interaction between time and group variables.

**Table 4:** LMM models for predicting WBC concentrations (K/uL).

	WBC (*n* = 91)				Neutrophils (*n* = 96)				Lymphocytes (*n* = 91)				Monocytes (*n* = 96)			
	B	SE	95% CI	*p*	B	SE	95% CI	*p*	B	SE	95% CI	*p*	B	SE	95% CI	*p*
Time ^a^	1.43	0.37	0.68, 2.18	0.00	1.17	0.29	0.60, 1.74	0.00	0.36	0.09	0.17, 0.54	0.00	−0.10	0.06	−0.21, 0.02	0.1
Group ^b^	0.83	0.51	−0.19, 1.84	0.11	0.24	0.37	−0.49, 0.97	0.52	0.33	0.16	0.01, 0.66	0.04	0.20	0.08	0.04, 0.35	0.02
Time*Group ^c^	−0.97	0.51	−1.99, 0.06	0.07	−0.72	0.40	−1.51, 0.07	0.07	−0.08	0.13	−0.34, 0.17	0.51	−0.10	0.08	−0.26, 0.05	0.2
Smoking	1.08	0.47	0.15, 2.01	0.02	-	-	-	-	-	-	-	-	-	-	-	-
Employment	-	-	-	-	-	-	-	-	−0.12	0.17	−0.45, 0.22	0.49	-	-	-	-
Gender	-	-	-	-	-	-	-	-	-	-	-	-	0.10	0.05	0.01, 0.19	0.03
Disease Stage	-	-	-	-	-	-	-	-	-	-	-	-	0.19	0.06	0.07, 0.32	0

B = Estimate of fixed effect, SE = standard error, *p* = *p*-value, CI = confidence interval, ^a^ Time = T0, ^b^ Group = WGJ intervention group, ^c^ the interaction between time and group variables.

**Table 5:** Clinical parameters summary.

Variable	Intervention (*n* = 50)	Control (*n* = 50)	*p*
Diarrhea grade I–II, N (%)	9 (18%)	9 (18%)	
Diarrhea grade ≥III, N (%)	8 (16%)	11 (22%)	0.47
Nausea and vomiting grade ≥II, N (%)	3 (6%)	8 (16%)	0.12
Number of hospitalizations during treatment, N (%)	11 (22%)	14 (28%)	0.64
Number of patients with relapse, N (%)	14 (28%)	9 (18%)	0.24
Overall survival 3rd year, N (%)	47 (94%)	42 (84%)	0.14

*p* = *p*-value.
